# The Importance of Neonatal Screening for Galactosemia

**DOI:** 10.3390/nu15010010

**Published:** 2022-12-20

**Authors:** Ioana Badiu Tișa, Anca Cristina Achim, Anamaria Cozma-Petruț

**Affiliations:** 1 Department of Mother and Child Care, “Iuliu Haţieganu” University of Medicine and Pharmacy, 2–4 Câmpeni Street, 400217 Cluj-Napoca, Romania; 2 Department of Bromatology, Hygiene, Nutrition, “Iuliu Haţieganu” University of Medicine and Pharmacy, 6 Pasteur Street, 400349 Cluj-Napoca, Romania

**Keywords:** galactosemia, newborn screening, galactose-1-phosphate uridyltransferase, galactokinase, UDP galactose-4-epimerase, galactose mutarotase, incidence, therapeutical approaches, galactose-restricted diet

## Abstract

Galactosemia is an inborn metabolic disorder caused by a deficient activity in one of the enzymes involved in the metabolism of galactose. The first description of galactosemia in newborns dates from 1908, ever since complex research has been performed on cell and animal models to gain more insights into the molecular and clinical bases of this challenging disease. In galactosemia, the newborn appears to be born in proper health, having a window of opportunity before developing major morbidities that may even be fatal following ingestion of milk that contains galactose. Galactosemia cannot be cured, but its negative consequences on health can be avoided by establishing precocious diagnosis and treatment. All the foods that contain galactose should be eliminated from the diet when there is a suspicion of galactosemia. The neonatal screening for galactosemia can urge early diagnosis and intervention, preventing complications. All galactosemia types may be detected during the screening of newborns for this disorder. The major target is, however, galactose-1-phosphate uridyltransferase (GALT) deficiency galactosemia, which is diagnosed by applying a combination of total galactose and GALT enzyme analysis as well as, in certain programs, mutation screening. Most critically, infants who exhibit symptoms suggestive of galactosemia should undergo in-depth testing for this condition even when the newborn screening shows normal results. The decision to enroll global screening for galactosemia among the specific population still faces many challenges. In this context, the present narrative review provides an updated overview of the incidence, clinical manifestations, diagnosis, therapy, and prognosis of galactosemia, questioning under the dome of these aspects related to the disease the value of its neonatal monitoring.

## 1. Introduction

Galactosemia is a disorder of carbohydrate metabolism, transmitted autosomal recessively, that affects newborns who are born asymptomatic, apparently well and healthy but who develop severe morbidity and sometimes even mortality after being breastfed or fed infant formula containing galactose. Galactose is an aldohexose that is essential for the galactosylation of endogenous and exogenous proteins, ceramides, myelin sheath metabolism, and energy production. Galactose is important for the human body, exhibiting numerous functions, as a key energy source in pre-weaning infants. Additionally, it has a significant structural role, especially in the early stages of development [1,2]. 

The inability to metabolize galactose leads to galactosemia [3,4]. Enzymes that metabolize galactose are active in the Leloir pathway, and they consist of galactose-1-phosphate uridyltransferase (GALT), galactokinase (GALK), uridine diphosphate (UDP)-galactose 4-epimerase (GALE), and galactose mutarotase (GALM). If any of these enzymes are deficient, galactose builds up, and the consequence is galactosemia [5,6,7]. 

Thus, several types of galactosemia can be distinguished: type I resulting from GALT deficiency, type II resulting from GALK deficiency, type III resulting from GALE deficiency, and type IV resulting from GALM deficiency, respectively. Galactosemia caused by GALT deficiency may be further classified into three phenotypes: classic galactosemia, clinical variant galactosemia, and biochemical variant galactosemia. Classic galactosemia is characterized by severe GALT deficiency, the activity of the enzyme either lacking or being very poorly detectable at the level of erythrocytes and the liver. While the clinical variant of galactosemia is linked to 1 to 10% residual GALT activity in erythrocytes and/or the liver, the biochemical variant is linked to 15 to 33% residual GALT activity in erythrocytes. The biochemical variant galactosemia includes the Duarte variant galactosemia [8].

In 1908, a newborn with galactosemia who experienced acute systemic toxicity was described for the first time. Over 25 years later, in 1935, the case of an infant with hypergalactosemia who responded favorably to a lactose-restricted diet was detailed [6]. Since then, ongoing research has been conducted to gain more insights into the molecular and clinical basis of this challenging pathology, with the ultimate goal of improving patient care worldwide. In recent decades, studies have also been conducted on cell and animal models, leading to an important advancement of knowledge [9,10].

The neonatal screening for galactosemia can urge early diagnosis and intervention, preventing complications. All galactosemia types may be detected during the screening of newborns for this disorder. However, the major target is GALT deficiency galactosemia, which is diagnosed by assessing blood galactose concentration and erythrocyte GALT enzyme activity and, for certain programs, by performing mutation screening [11,12,13]. 

## 2. Methodology

We performed a narrative literature review, based on the relevant scientific articles written in English, which have been identified in the PubMed, EMBASE, and Scopus databases. Keywords such as “galactosemia”, “newborn screening”, “galactose-1-phosphate uridyltransferase”, “galactokinase”, “UDP galactose-4-epimerase”, “galactose mutarotase”, “incidence”, “therapy”, and “galactose-restricted diet” were used individually or in combination within the literature search. We mainly included original research articles but selected also several high-quality review articles that contained the most up-to-date information. Generally, we focused on recent publications but did not set limits concerning the date of publication.

## 3. Results

### 3.1. Incidence

The overall incidence of galactosemia varies by race and ethnicity. It is the highest among Caucasians and ranges between 1:16,000 and 1:44,000 among infants in the United Kingdom and Ireland. Ireland has a high incidence of classical galactosemia of 1:16,476, especially in the Traveller ethnic group (1:430). On the other hand, Sweden has a relatively low frequency (1:100,000). The most common pathogenic clinical variant p.Ser135Leu/Ser135Leu in the African American and South African genotypes has an estimated prevalence of 1:20,000. The incidence of type I galactosemia ranges from 1:40,000 to 1:60,000. An increased incidence is shown among individuals of Irish origin (1:24,000), whereas the lowest incidence is reported among people of Swedish origin (1:100,000) and Japanese origin (1:788,000) [12,14]. In the United States of America, the prevalence has been reported at values between 1:30,000 and 1:60,000 live births [15,16]. The prevalence of GALK deficiency ranges from 1:22,000 to 1:50,000, with the Roma populations of Bulgaria and Bosnia experiencing the greatest rates. People of African and Asian heritage appear to have a relatively low incidence of the condition. In the population of Western Europe, the incidence of galactosemia ranges between 1:23,000 and 1:44,000 [17,18,19].

Classical galactosemia (GALT) attributed to a CG genotype with two trans pathogenic variants is present in 1:53,554 newborns in the United States of America. As concerns the total GALE deficiency, until now, it has been very rare for this deficiency to be diagnosed. The epimerase deficiency established by neonatal screening, including mainly peripheral or intermediate forms, is 1:70,000 in European infants and 1:6,700 in African American infants, respectively. Due to incomplete identification, it is challenging to determine the prevalence of Duarte variant galactosemia. However, it is estimated that Duarte variant galactosemia currently affects around 10 times as many newborns in the US as classical galactosemia [15,19].

### 3.2. Clinical Manifestations and Diagnosis

Galactosemia includes a rare group of hereditary disorders of galactose metabolism. To date, four types have been described, each affecting a different step in the main route of galactose disposal: type I, resulting from GALT deficiency; type II, resulting from GALK1 deficiency; type III, resulting from GALE deficiency; and the recently described type IV galactosemia, resulting from GALM deficiency [20,21].

GALT deficiency results in classic galactosemia, which is a rare, serious, life-threatening condition, in which symptoms most often begin in the second half of the first week of life. The newborn ingests lactose from breast milk or infant formula, lactose being a disaccharide that comprises equal parts of the monosaccharides glucose and galactose. If GALT is absent, the patient is unable to metabolize galactose-1-phosphate, which accumulates at liver, kidney, and brain levels, causing damage to these organs. Lesions can also be initiated before birth, following the exposure of the fetus to the galactose, when the carbohydrate originating from the diet of the heterozygous mother or from fetal endogenous synthesis is absorbed through the placenta. Relative control of galactose-1-phosphate levels does not always show a direct association with long-term outcomes [22,23].

GALT deficiency and associated clinical signs and symptoms that may appear in the first days or weeks following birth are also monitored in the newborn. Clinical manifestations of this deficiency may include vomiting, jaundice, hepatomegaly, hypoglycemia, lethargy, refusal to eat, weight gain, and seizures. Partial transferase deficiency is more common than classic galactosemia, is asymptomatic, and is diagnosed by screening the newborn based on a determination of moderately increased blood galactose and/or low transferase activity. The condition should be taken into consideration for the newborn who is underweight or has any of the above signs and symptoms [17,21,24].

The initial diagnosis of galactosemia is established by showing the presence of reducing substances in a number of urine samples taken while the child is consuming breast milk or lactose-containing infant formula. Galactose can be identified in the urine if the patient is fed with lactose several hours before the analysis and only if the patient does not vomit excessively.

Several tests can be used to diagnose classic galactosemia. Testing the urine for the presence of reducing substances can be informative. However, this type of test lacks sensitivity and specificity, leading to false positive results in the case of fructosuria, lactosuria (caused by intestinal lactase deficiency), or conditions that affect the clearance of blood galactose, such as severe liver disease or antimicrobial therapy. On the other hand, if the infant is receiving intravenous nutrition, galactosuria may be absent, thus leading to false negative results [25,26].

Measuring, in the blood and/or in the urine, the galactose metabolites, such as galactose 1-phosphate and/or galactitol, seems to be a more precise method. Samples collected after transfusion should be noted on the specimen card and repeated as recommended by the country protocol [27,28,29].

The diagnosis of classical galactosemia, including a partial deficiency, is then confirmed by a laboratory test for galactosemia to detect a high concentration of erythrocyte galactose-1-phosphatase, urinary galactitol, and by the identification of bi-allelic pathogenic variants in the GALT gene. A new method uses non-radioactive ultraviolet light and high-performance chromatography to detect in an accurate manner the GALT levels in erythrocytes [12,13].

Infants who continue to receive breast milk or infant formula containing lactose may develop liver failure, ascites, cirrhosis, splenomegaly, cataracts, vitreous hemorrhage, and mental retardation. Galactosemia patients are more likely to develop an infection with *Escherichia coli*, which can evolve into neonatal sepsis, this infection preceding the diagnosis of galactosemia. The complete removal of lactose from the diet and the use of soy milk formulas leads to the improvement of acute symptoms. If left untreated, death can occur within a few days due to liver failure, kidney failure, or sepsis with *Escherichia coli* [30,31,32].

GALK is the enzyme that accelerates the phosphorylation of galactose, and the major metabolites that accumulate are galactose and galactitol. GALK is known to be encoded by two genes: GALK1 on chromosome 17q24 and GALK2 on chromosome 15q21.1–q21.2. The child with GALK deficiency is asymptomatic but frequently develops cataracts. According to recently published data, the phenotypic manifestations of GALK1 deficiency may also include an increase in transaminases during the neonatal period, bleeding diathesis, and encephalopathy [33]. The diagnosis of GALK deficiency is established by showing a lack of GALK activity in erythrocytes or fibroblasts. Transferase activity is normal, and the galactose dietary restriction is used as the therapeutic approach [34,35].

GALE deficiency has two distinct variants, one benign and one severe. The benign variant is accidentally diagnosed by screening programs for newborns, the affected individuals exhibiting no symptoms due to the fact that enzyme deficiency is limited to leukocytes and erythrocytes. Nevertheless, it has been demonstrated that GALE deficiency is a continuum disorder rather than a binary condition [36]. The benign type of GALE deficiency does not require treatment. The severe variant results from generalized GALE deficiency. Clinical manifestations are comparable to those in GALT deficiency, and they include nervous deafness and hypotonia. Clinical symptoms are improved by restricting galactose in the diet. Despite the rarity of the severe type of galactosemia, this type should be taken into consideration in any patient with symptoms and measurable galactose-1-phosphate who has normal transferase activity. Abnormally accumulated metabolites are comparable to those in GALT deficiency. Nevertheless, uridine cell diphosphate (UDP) galactose is also increasing [37,38]. Furthermore, GALE deficiency has recently been associated with thrombocytopenia [39].

Erythrocyte epimerase analysis is used to validate the diagnosis of GALE deficiency. Galactose dependence and inability to synthesize UDP galactose from UDP glucose are features present in patients with the severe form of epimerase deficiency. Since many structural proteins in the nervous system contain galactose as an essential structural element, patients are treated with a galactose-restricted diet rather than a galactose-free diet. Treatment is not necessary for infants with a mild form of epimerase deficiency. Within a few weeks following the diagnosis, it is recommended for urine samples to be analyzed for reducing substances while the child continues to be fed lactose-containing formula [40,41]. The GALE gene is located on chromosome 1 at 1p36. Epimerase activity in erythrocytes can be measured to identify carriers. The diagnosis of the severe form of epimerase deficiency before birth can be made by performing an enzymatic assay of cultured amniotic fluid cells [6,42].

As concerns type IV galactosemia, this disease is caused by biallelic mutations in the GALM gene, leading to decreased activity of the GALM enzyme, which supports the equilibrium between the a- and b-anomers of D-galactose. Type IV galactosemia has an estimated prevalence of 1:228,411 in all populations, 1:80,747 in the population of Japan, and 1:10,388 in the population of Africa, respectively [43].

Type IV galactosemia is associated with elevated blood galactose levels and with an absence of changes in galactose 1-phosphate concentrations, respectively [44]. Type IV galactosemia seems to show symptoms that are comparable to those in type II galactosemia. This may lead to the risk of misdiagnosing type IV galactosemia as type II. To avoid such circumstances, the sequencing of GALK and GALM genes is important [44]. Regarding the long-term consequences of type IV galactosemia, they remain to be identified, as the disease has only relatively recently been discovered. The therapeutical approach in type IV galactosemia involves the dietary restriction of galactose [45].

### 3.3. Treatment and Prognosis

A galactose-restricted diet has been shown to be very effective in treating life-threatening neonatal manifestations and has been the cornerstone of treatment for galactosemia. This highlights the importance of studies to renew evidence-based practice guidelines and the role that healthcare plays in caring for these patients throughout their lives [46,47].

Despite evidence questioning the benefits of strict dietary restriction, lifelong galactose restriction and long-term follow-up remain the standard of care, while the restriction for the Duarte variant remains controversial. Indeed, a recent study demonstrated that regardless of whether children with Duarte galactosemia consume milk or low-galactose formula during infancy, they are not more likely to experience acute problems or early childhood developmental issues that need intervention [48].

Genetic counseling should be provided to assess genetic testing possibilities, in order to establish a prognosis, guide therapy, and diminish the risk of recurrence for the potential upcoming pregnancies [49].

Galactose should be eliminated from the diet when galactosemia is first suspected, with the cessation of breastfeeding and the use of lactose-free milk replacers such as casein hydrolysates (e.g., Nutramigen^®^, Alimentum^®^, Pregestimil^®^) or soy-based formulas (e.g., Isomil^®^, Prosobee^®^) [8]. As concerns soy infant formulas, there has been much debate regarding their safety over long-term use, as they contain plant isoflavones that may affect hormonal status in children [50]. Nevertheless, a recently published systematic review and meta-analysis did not identify any correlation between soy-based infant diets and the initiation of puberty in male and female children [51]. Furthermore, both casein hydrolysates and soy-based formulas still have trace amounts of galactose. In this context, several studies have reported that the complete removal of galactose from the diet by using a formula that contains neither free nor bound galactose (Neocate^®^) leads to a considerably faster reduction in the high erythrocyte galactose-1-phosphate values that are identified during infancy in children with galactosemia [52,53,54].

Galactose removal from the diet and proper calcium intake improve weight gain, prevent liver and kidney failure, and allow the regression of cataracts, with most patients remaining with no vision impairment [35,55].

The prognosis for galactosemia is improved by an early diagnosis and a consequently early initiation of treatment. In evolution, however, patients show ovarian failure with primary or secondary amenorrhea, and hypogonadism is documented in 80% to 90% of women with classic galactosemia. Other manifestations are delayed development and learning difficulties that worsen with age as well as a decrease in bone mineral density, and most patients having speech disorders, impaired motor function, and balance disorders [56,57,58].

To date, there is no knowledge about interventions that could stop the emergence of the long-term complications observed in patients with classic galactosemia, such as the case of the development of primary ovarian insufficiency [59].

The occurrence of these burdensome long-term health issues has raised a great interest in designing therapies that could contribute to their prevention. Several novel therapeutic approaches currently being investigated aim for the restoration of the activity of the GALT enzyme and include GALT gene therapy [60,61,62], mRNA therapy [63], and pharmacological chaperones [64]. Other potential therapies focus on influencing the cascade of events in galactosemia and include GALK1 inhibitors [65,66], aldose reductase inhibitors [67], and endoplasmic reticulum stress reducers [68,69].

### 3.4. Neonatal Screening

Neonatal screening for classical galactosemia may protect newborns from life-threatening complications in early life. Nevertheless, it does not prevent the development of long-term consequences, whilst the complicated pathophysiology of this rare disease remains poorly comprehended [12,25,70].

The availability of newborn screening for galactosemia makes it possible to identify and treat patients much earlier than in the past. Many nations around the world evaluate galactosemia either on a governmental or a non-governmental basis. In Ireland, screening of newborns for classical galactosemia has been performed since 1972. On the other hand, galactosemia is a rather uncommon condition in Sweden, and yet the screening program for newborns includes this disease [16,71,72].

There has been discussion about newborn screening for classic galactosemia for a long time, but there is still no agreement in place. Several newborn screening programs have excluded galactosemia on the basis of two main justifications: the costs are too high compared to the incidence of the disease and the fact that some long-term issues arise even with early intervention. The whole reversal or the avoidance of galactosemia morbidities (e.g., primary ovarian insufficiency, speech difficulties) is not guaranteed by a rigorously adopted galactose-free diet. For example, even with early intervention (dietary galactose limitation), the long-term consequences of classical galactosemia in patients from Ireland have been reported to be important: 30.6% of patients older than 6 years had IQs under 70, 49.6% of patients older than 2.5 years had speech or language issues, and 91.2% of female patients older than 13 years suffered from hypergonadotropic hypogonadism that may further affect fertility. This type of data provides potential justification against mass neonatal screening, rather supporting an at-risk population screening. However, there have been recommendations that all samples with elevated phenylalanine levels be tested for galactosemia as an additional test [69,73,74,75].

## 4. Discussions

The occurrence of innate metabolic errors, the importance of early diagnosis after the birth of the newborn, and genetic counseling are strong arguments for universal screening of all newborns. Even with screening, a child with galactosemia may be omitted. Infants who receive a low-dose galactose formula at screening and those who have received a blood transfusion may obtain a false negative result. A thorough examination of the test result, patient history, and nutrition at the time of the test guarantees a correct interpretation. Most notably, newborns with symptoms suggestive of galactosemia should be tested even when neonatal screening shows normal results. A routine galactosemia gene panel test is available in Norway, Denmark, and Scotland to help in diagnosis [12,48,75].

According to newborn screening in the US, the incidence of the condition is at 1:47,000 live births. There are different enzymatic variants of galactosemia. By performing a direct enzymatic assay on amniocytes or chorionic villi, carrier testing and prenatal diagnosis can be performed. DNA testing is another option. Using an enzymatic assay of cultured amniotic fluid cells, the severe type of epimerase deficiency can be identified during pregnancy. The Duarte variant is the most common, having a frequency of 12% and involving a substitution of a single amino acid (p.N314D) and a decrease in the activity of the red blood cell (RBC) enzyme to 50% of normal but without clinical significance. More than 230 pathogenic variants with transferase deficiency have been identified. In the white race, 70% of the alleles are represented by p.Q188R and p.K285N and are linked to severe forms, while in the color population, 62% of the alleles are represented by the variant p.S135L, a variant mainly associated with mild disease. Among neonates with Duarte variant galactosemia, certain patterns involving differing prevalence based on race are evident. Thus, infants of European heritage are more likely to develop Duarte variant galactosemia than infants of African, African American, or Asian descent. These variations are similar to the variations identified at the level of these populations in the prevalence of variant D2 and/or other known pathogenic GALT variants [44,45,76].

Neonatal screening for galactosemia is performed in several countries in Europe, such as Austria, Germany, Hungary, Ireland, Sweden, Switzerland, and the Netherlands, while countries such as Turkey, Italy, and Belgium have pilot programs [15,45]. Newborns in Ireland are screened within 72 to 120 h after birth, while all high-risk Traveller newborns are evaluated on the first and second day after birth. Other European countries, such as France and Romania, do not provide neonatal screening, whereas Norway, Denmark, and Scotland no longer run their programs [30,76,77]. In Romania, there is not yet a database of patients with galactosemia, and neonatal screening is performed only for phenylketonuria and hypothyroidism.

In Canada, only two out of ten provinces provide universal screening for galactosemia, compared to all 50 states in the United States. As a screening approach, a positive result can necessitate repeat testing to confirm the diagnosis, and the turnaround time for test results varies from country to country. Sometimes, the clinical manifestations are severe enough before the results of the newborn screening are ready. When a patient has symptoms that suggest a metabolic condition, but the screening is negative, sending the patient to the genetics center for additional testing is justified. A better prognosis has a greater likelihood of occurring with early diagnosis and treatment. However, as galactosemia is a rare, non-specific disease, it requires strong clinical suspicion for investigation and diagnosis [15,78,79,80].

The International Galactosemia Network created the GalNet registry, a patient database for the condition, in 2014. The objective was to establish a specific registry for classic galactosemia, to monitor the pathology on a long-term basis and to assess the safety and efficacy of various treatments using data from a large number of patients. The American College of Medical Genetics Task Force recommended for newborn screening to also include galactosemia along with other metabolic disorders [33].

As concerns neonatal screening in galactosemia, there is still no unified opinion. The screening of a newborn for this disorder involves taking a capillary blood sample within the first 72 h of birth and measuring the level of galactose and/or GALT enzyme activity. Galactosemia screening has been incorporated into national programs in many countries. However, due to the high number of false positive tests, it is not as common in Europe [25].

There is clear evidence that the screening of newborns for galactosemia reduces the risk of suffering, morbidity, and mortality. Thus, despite the hurdles, difficulties, and complexity, screening for galactosemia is advised. The careful clinical assessment of the newborn and selective screening allow identifying most cases of severe galactosemia [70,75].

The arguments in favor of newborn screening have given rise to optimism for the development of early detection and fast treatment. In the diagnosis of galactosemia, the universal screening enables early dietary changes to prevent life-threatening clinical manifestations caused by high quantities of galactose (example: liver failure and its complications). According to research, mortality was statistically reduced more than ten times over a period of ten years by newborn screening for galactosemia. More precisely, mortality was reduced from 4.6 to 0.3. Additionally, there are cases in which neurological damage can be prevented, which is also crucial given that it has been shown that the expense of caring for a child with mental retardation might surpass USD 1 million. It is a reality that the costs of galactosemia screening alone could exceed the benefits, but on the other hand there is the option of including the screening for this disorder in a newborn screening program that already exists, an approach that has been shown to have net benefits with a benefit/cost ratio of two [81].

The early treatment in galactosemia is not always effective in preventing neurological issues (e.g., memory difficulties, tremors, language and speech defects) and can still evolve with ovarian failure in female patients. However, early detection and treatment are considered to reduce mortality, early morbidity, and complications, preventing disability and enhancing the quality of life [82,83,84].

The annual expenses of the health system for patients with galactosemia are high, particularly when considering the forms showing severe neurological damage and requiring intervention for cataracts and/or hormone replacement treatment. Furthermore, looking from a long-term perspective, it has been suggested that funding full neonatal screening may generate financial savings for society. For instance, a study from Iran showed that the implementation of neonatal screening for galactosemia lowered the cost burden of the disease by two-thirds [25,71,73,81].

The arguments against universal screening for galactosemia are related to the costs it involves, calculated right from the initiation of screening planning and related to the training of professionals, the implementation of the screening system, the questionable accuracy of newborn testing, the complexity of the processes of counseling parents, and patient confirmation. Likewise, the large regional difference in the disease’s incidence weighs heavily in a country’s decision on whether to perform the screening or not [73,75,81].

## 5. Future Recommendations

The logistics of neonatal screening are expected to be facilitated by recent developments in biotechnology and communication. For instance, there is a special need to optimize newborn screening for classical galactosemia, which still shows a relatively high false positive rate [71]. Indeed, recent studies show opportunities regarding the use of next-generation sequencing in the neonatal screening of the inborn errors of metabolism. Next-generation sequencing is expected to be associated with a lower risk of false positive results [85].

Furthermore, it is important to develop personalized nutritional approaches in galactosemia. This idea is supported by the example of variant patients with an activity of the erythrocyte GALT enzyme of up to 10%, in which case it must be clarified to what extent a severe restriction of galactose is really needed, particularly since over-restriction of galactose in the long-term can be harmful [71,86].

Worldwide, many organizations work together to support neonatal screening programs, and in countries where there is a population at risk of galactosemia, the neonatal screening of the disorder must unquestionably be considered [70].

## 6. Conclusions

Galactosemia is a rare, non-specific disease, therefore requiring strong clinical suspicion for investigation and diagnosis. It is now possible to diagnose and treat patients considerably earlier than before thanks to the availability of neonatal screening for galactosemia.

Neonatal screening is especially needed to detect classic galactosemia. If the diagnosis is not established immediately after birth, to consequently restrict galactose sources from the diet, the injury to organs such as the liver, kidneys, and brain becomes highly severe and irreversible, sometimes even fatal. Early diagnosis and treatment in galactosemia lead to a higher chance of a better prognosis, whereas infants and children that are left untreated suffer from major health consequences, such as intellectual disability, behavioral issues, poor growth, deficient development, nutritional deficiencies, and sequelae requiring advanced hospital care.

The implementation of a screening program for galactosemia in newborns varies from country to country and necessitates taking into account a variety of factors related to early diagnosis, such as the prompt introduction of the diet therapy but also the cost–benefit ratio of this screening.

## Data Availability

Not applicable.

## Abbreviations

CG—classical galactosemia; GALE—uridine diphosphate-galactose 4-epimerase; GALK—galactokinase; GALM—galactose mutarotase; GALT—galactose-1-phosphate uridylyltransferase; UDP—uridine diphosphate.
